# Measuring Research Engagement among Previously Incarcerated Persons and Other Carceral Stakeholders while Implementing Wastewater-based Surveillance for SARS-CoV-2 in Jails: A Mixed-Methods Study

**DOI:** 10.21203/rs.3.rs-7456979/v1

**Published:** 2025-10-14

**Authors:** Victoria M. Brown, Alexandra E. Kauffman, Amadin A. Olotu, Saachi Kumar, Rachel A. Boehm, Lindsey R. Riback, Chad J. Zawitz, Melody S. Goodman, Matthew J. Akiyama, Anne C. Spaulding

**Affiliations:** Emory University RSPH; Emory University RSPH; Emory University RSPH; Emory University RSPH; Emory University RSPH; Albert Einstein College of Medicine; Cook County Health; New York University School of Global Public Health; Albert Einstein College of Medicine; Emory University RSPH

**Keywords:** Jail, Participatory Research, Research Engagement Survey Tool (REST), Implementation Science, Stakeholder Engagement, Research Engagement

## Abstract

**Introduction:**

Engaging persons with lived experience of incarceration (PLE) in projects to improve carceral healthcare is recognized as important but is understudied. We leveraged an implementation project seeking to improve carceral infection control using SARS-CoV-2 wastewater-based surveillance (WBS) to study the engagement of diverse carceral stakeholders including PLE on implementation teams.

**Methods:**

From April 2022 to December 2023, WBS Implementation teams were formed at four US jails. These included diverse carceral stakeholders: custodial and medical leadership, facilities management, and medical staff positions, along with PLE. Focus group discussions (n = 12) and key informant interviews (n = 6) facilitated internal and external collaboration between teams. The study team shared qualitative findings of PLE and carceral personnel between the two groups. We used Rapid Assessment Processes for qualitative thematic analysis to understand key themes related to dynamics of engagement. We used the Research Engagement Survey Tool (REST) to quantify the quality and quantity of stakeholder engagement.

**Results:**

Qualitative data demonstrate carceral personnel support for including PLE in decision-making improved over the project once PLE feedback was shared, although PLE expressed that the partnership ideally would have been on more equal footing. PLE perspectives were deemed useful to carceral personnel and the development of a sustainable system to incorporate resident feedback specific to mitigation efforts was a priority of both carceral personnel and PLE. Medical leaders identified barriers in engaging staff in WBS due to the hierarchal structure of decision-making. On a 1–5 Likert scale, REST scores aligned with qualitative data indicating lower engagement for PLE (mean 3.2 versus 3.9 for quality and 3.3 versus 4.1 for quantity of engagement). PLE had consistently lower REST scores than carceral personnel across all measured engagement principles.

**Conclusion:**

This study brought disparate carceral stakeholders together to support the implementation of jail-based WBS and highlighted disparities in collecting and incorporating PLE feedback into jail infection control. Strengthening collaboration between the PLE and carceral personnel would ensure carceral health systems benefit from all perspectives.

## Introduction

The burden of the SARS-CoV-2 pandemic was particularly pronounced in the US carceral system (Craig, 2023) (Akiyama et al., 2020). Some carceral facilities used wastewater-based surveillance (WBS) to monitor the burden of SARS-CoV-2 levels in their facilities, a strategy that had been used institutionally on college campuses and communally using municipal wastewater treatment systems (Harris-Lovett, 2021) (Saber, 2024 Apr). Though feasible and effective, the introduction of WBS in carceral settings benefits from a rigorous and sustainable implementation strategy.

Challenges in harmonizing mitigation protocols among custodial and medical sectors, difficulties in mass testing, low resources, and mistrust of jail officials involved in health messaging and protocol enforcement have been well-documented in carceral settings (Brown, 2024; Riback, 2023). WBS has been recognized as a cost-effective tool to alleviate some of these challenges by complementing often limited clinical surveillance for COVID-19 and informing mitigation strategies prior to case confirmation with its ability to detect pre-symptomatic and asymptomatic SARS-CoV-2 infections (Buitrago-Garcia et al., 2020; Harris-Lovett, 2021; Kennedy et al., 2024). The Conducting Correctional COVID Research and Implementing Novel, Ethically Sound, Sustainable Surveillance Systems (CRAINES) project sought to address the challenge of WBS implementation by creating implementation teams in four U.S. jails. Teams were composed of various “carceral personnel” (CP)— a term defined as stakeholders employed within the carceral system as custodial/medical leadership, facilities management, or medical staff— and persons with lived experience of incarceration (PLE) at the facility (Appendix 1). WBS has previously been demonstrated as an acceptable strategy for infection surveillance among PLE (Brown, 2024). Additionally, PLE offered perspectives on operationalizing WBS in jails, including ways to improve health messaging for mitigation efforts, benefits and challenges associated with implementing WBS, and the acceptability (or lack thereof) of monitoring for other pathogens and detectable agents such as MPOX, influenza, HIV, and illicit drugs (Brown, 2024).

Stakeholder engagement is becoming increasingly well-established as an important element for implementation research and public health practice (Viswanathan, 2004). Although partnerships between those developing and implementing policies and those affected by them have been demonstrated to improve the success and sustainability of interventions, stakeholder-engaged research with PLE has not been well studied in the US criminal legal system (Wurcel, 2022). This is due, in part, to sociopolitical factors and negative stigma associated with incarcerated persons, further contributing to the marginalization of this population in public health and participatory research.

Utilizing the teams consisting of PLE and CP from CRAINES, we sought to assess partner engagement to fill knowledge gaps in carceral health participatory research. In addition to qualitative research approaches (Khodyakov et al., 2013; Martinez, 2019), we also utilized the Research Engagement Survey Tool (REST) as a quantitative measure of stakeholder engagement in this project. Principles included in the REST are derived from foundations of community-based participatory research and community engagement (Goodman et al., 2022; Goodman, 2020; Thompson, 2020). The REST has been validated, (Goodman et al., 2022) and its use is expanding in participatory research (Berrios, 2024; Guilamo-Ramos, 2024; Hoke A.M., 2023). We hypothesized that utilizing a quantitative measure along with identifying qualitative themes shared by PLE and CP would allow us to further explore the intricacies of WBS implementation among the teams of interdisciplinary stakeholders included in CRAINES.

Amid calls for participatory research to inform carceral healthcare delivery (Akiyama et al., 2021; Wurcel, 2022), we sought to leverage the CRAINES project adhering to principles of community engagement and collaboration to support inclusion of PLE as stakeholders on implementation teams. In this manuscript, we report the findings of our evaluation of the engagement of PLE in the implementation process with traditional carceral stakeholders.

## Methods

In April of 2022, WBS Implementation teams were formed at the jails of Cook County (IL), Fulton County (GA), Washington DC, and Middlesex County (MA). The four institutions varied in size and architectural layouts. Cook County as the largest facility (Average Daily Population 6000) and Middlesex as the smallest facility (Average Daily Population 825). Each facility was at a different stage of adopting WBS into their infection control (IC) systems and sought to promote WBS implementation (Kennedy et al., 2024)

The CRAINES project created implementation teams of disparate carceral stakeholders at each facility with members being either CP employed in the jail system — such as custodial and medical leadership, facilities management, and medical staff — or PLE of incarceration at the facility. Team members were referred by site leads — individuals who served as investigators, staff, or persons with both roles— and were approached face-to-face or electronically. Site leads purposively offered referrals to individuals who had a stakeholder role predetermined by study design (Appendix 1). Site leads identified 7 PLE to refer through reentry programs, community clinics, or advocacy groups. One of the 7 PLE recruited the eighth peer. Team members were accepted on a rolling basis. Referred CP who chose not to participate cited competing workload demands. Two sites chose to have limited CP participation on implementation teams at their discretion. Over the span of the project, there was CP turnover due to competing work demands and employment status. Teams ranged from 6–12 members, with all sites having two recently released PLE who were incarcerated for a month or more at the corresponding jail site during the SARS-CoV-2 epidemic. Most PLE identified as male (87.5%) and Black (87.5%) (Table 1).

In April 2022, two meetings were held to introduce the study team, outline project goals, and define roles of the implementation teams for CP. They were encouraged to collaborate internally, independent from study activities, to accomplish their facilities’ WBS goals. One meeting with PLE from all sites was held to introduce the study team, outline project goals, and describe WBS and participatory research. As part of study design, PLE and CP implementation team members did not directly communicate due to existing power dynamics between the two. Instead, the study team shared information between CP and PLE over the course of the project.

### Qualitative Data Collection

Project activities included focus group discussions (FGDs) and key informant interviews. The lead author, VMB, a female project coordinator trained to the master’s level in public health with training in rapid qualitative analysis and Consolidated Framework for Implementation Research (CFIR) constructs partnered with one other author of similar backgrounds to conduct each of the FGDs and interviews and take field notes. Questions were presented via show cards for participants. 60 to 90-minute FGDs and interviews were conducted between May 2022 and December 2023 and recorded and transcribed over Zoom with hybrid participation options available for some. Only the lead author, co-investigators and participants attended. Guides were developed using the CFIR framework (Damschroder, 2022). FGDs were organized as either site-wide— in which participants had shared affiliation with a jail site, or cross-site—in which participants represented different jail sites. Site-wide FGDs aimed to identify gaps in WBS implementation specific to a jail, while cross-site FGDs aimed to foster the sharing of practices between jails. Cross-site FGDs participants were selected based on their discipline or role within their jail, in which participants held similar positions on the implementation team. Custodial leadership, medical leadership, and PLE all participated in cross-site FGDs. Key informant interviews were conducted with PLE to gain further insight into best strategies for health messaging for WBS, improving protocol adherence, and expanding WBS to other pathogens. Jails were encouraged to continue their advancement of WBS outside of study activities and did so through their own independent internal meetings over the course of the project.

Project activities were organized into two phases. Phase 1, representing all qualitative data collected prior to the sharing of PLE perspectives with CP and phase 2, after PLE input was shared via infographics and PowerPoint presentation using data collected in Phase 1 from PLE FGDs and key informant interviews presented elsewhere (Brown, 2024). In phase 2, CP were able to discuss and reflect on these findings and PLE were able to respond to their reflections.

**Figure 1.**
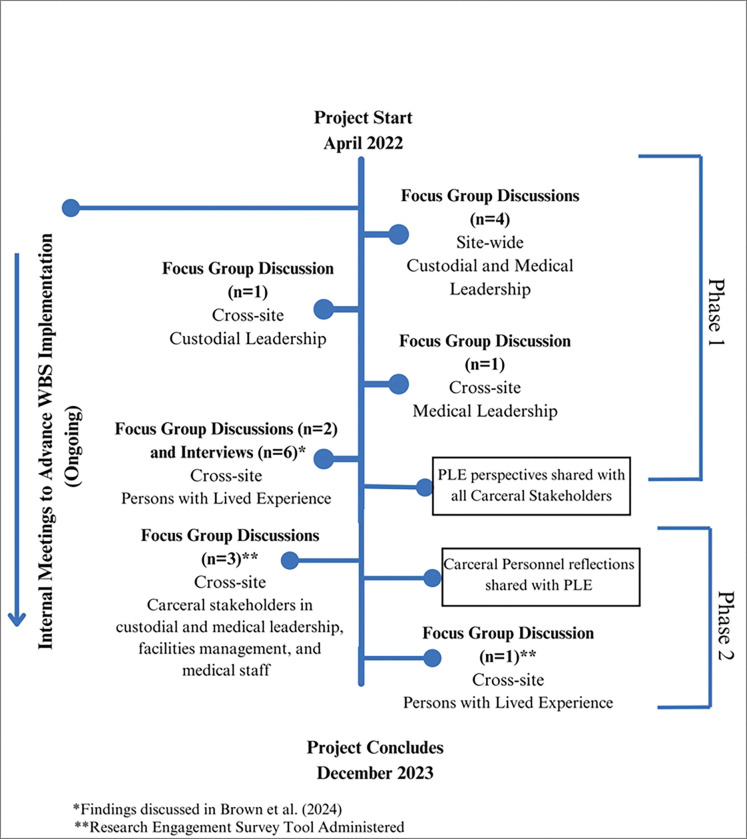
Timeline of Project Activities in CRANES Study, 4/2022–12/2023

Figure 1 illustrates the twelve total FGDs and six key informant interviews held. FGDs and interviews were transcribed; transcripts were not shared with participants.

We used Rapid Assessment Processes (RAP) for qualitative thematic analysis using a deductive and inductive approach. Two independent coders from the team of coders (VMB, AEK, AAO) reviewed and summarized each transcript using a structured analysis template organized from a priori themes derived from the CFIR to code transcripts. Themes that emerged inductively during the analysis were also recorded. Templates, including extracted quotes, were then iteratively reviewed by the qualitative study lead (MJA) and the rest of the team in periodic study meetings to refine themes, ensure alignment, and resolve discrepancies until data saturation was met. Final templates were used to consolidate themes with core qualitative investigators. To maintain the anonymity of participants, we present quotes by the discipline in which they serve on the team and not by the site in which they work or were incarcerated. We used the Consolidated Criteria for Reporting Qualitative (COREQ) checklist to guide the qualitative portion of our analysis (see Appendix 2).

### Quantitative Data Collection

At the conclusion of phase 2, REST assessments were administered to all implementation team members to assess the perceived quality of engagement between carceral stakeholders and PLE. The eight engagement principles (EPs) of REST are listed in Table 2.

We used the comprehensive version of this tool, which includes 32 items split among the 8 EPs (Appendix 3). We chose to omit EP 7 since all partners understood dissemination would be the role of the study team. Twenty-nine remaining items on the REST were rated for quality — how well the partners leading the research do the following, and quantity — how often the partners leading the research do the following. Quality is rated on a 5-point Likert scale ranging from 1 (poor) to 5 (excellent). Quantity is rated on a 5-point Likert scale ranging from 1 (never) to 5 (always). Both scales include the option of not applicable. Mean scores were calculated for quality and quantity scales and compared between carceral stakeholders and PLE.

## Results

### Qualitative Results: Synthesis and Interpretation

In our FGDs and interviews with PLE and CP, we sought to understand different perspectives regarding WBS, COVID mitigation protocols, and collaboration between implementation team members. In this manuscript, we organize their perspectives on the partnership between diverse stakeholders into two key themes: the perceived utility of including PLE as stakeholders and the mechanisms of collaboration between PLE and CP.

### a. Perceived Utility of Including PLE as Stakeholders

In phase 1 FGDs, we asked carceral leaders, of both the custodial and medical sectors, to comment on the inclusion of PLE on implementation teams. While open to participating as members of the same implementation teams, some carceral leaders questioned the utility of including PLE. Although carceral leadership understood the importance of improving the health conditions of residents, including PLE at their respective facilities was a novel concept to leadership and one deemed to be of potentially low impact.

“I don’t think the involvement of persons with lived experience would have much of an impact [on WBS adoption]… As long as our senior leadership are invested in it, it’s going to happen.”

— Custodial Leadership

CP also expressed that information on WBS was not likely to be a priority for residents. CP expressed that residents would have higher priorities than understanding the degree to which COVID is circulating in their facility.

“Worrying about COVID and things like that is the least of their worries; getting something in their stomach, finding a warm place to sleep…A lot of people don’t want to hear about that [COVID-related WBS].”

— Custodial Leadership

Such sentiments were also expressed by some medical stakeholders who did not hold ultimate policy decision-making agency in their day-to-day roles. Their reasoning for this centered on the lack of agency PLE and non-managerial staff have in policy changes, which historically come from their respective Sheriff’s Office. Medical leadership felt as if protecting staff and residents was their job, and their efforts were better concentrated among decision-makers. They expressed beliefs that WBS results were more important to staff than residents, who would benefit from downstream effects of mitigation protocols rather than WBS data or processes themselves.

“WBS doesn’t have an effect [on what] happens inside the jail… We’re like on an island, And the rest of the world is the ocean. We’re detecting what’s in the ocean…The people on the island are pretty much oblivious to it.”

— Medical Leadership

“The way we are collecting the data doesn’t really apply to any applicable use in prevention…that doesn’t contribute to anything but potentially causing panic…Data without context is useless.”

— Medical Leadership

Some described the hierarchal decision-making of their jail as a barrier in educating the greater jail community on WBS processes. Such structured divisions between decision-makers and staff was also highlighted as an important aspect of successful WBS implementation.

“I have to go through the higher ups, and if they say we’re not going to talk about it, we’re not going to do a talk on it, we’re just not going to do it”

— Medical Leadership

“Whereas I do really need facilities, right? To be excited by wastewater testing. But I really need the director of facilities.”

— Medical Leadership

On the other hand, PLE felt as though their perspectives on the implementation team was invaluable. Many expressed their desire for improving infection control began during their incarceration where they experienced rigorous COVID-19 mitigation protocols such as isolation, quarantine, and mass-testing.

PLE felt their experience with such protocols provided them with a perspective that could improve the roll out of future infection control programs including input on the best modes, methods, and communicators of health messaging, the expansion and accessibility of health education, and general feedback of how the culture of a jail can influence protocol adherence. Most PLE articulated support of robust infection control programs and receptiveness to expanding WBS to similar infectious agents. These findings are presented in greater detail elsewhere (Brown, 2024).

In phase 2, after PLE perspectives were shared, CP were able to reflect on the involvement of PLE. Some CP expressed surprise that PLE articulated enthusiasm and thoughtful suggestions about WBS, in which they assumed that lack of agency would deter PLE from having an interest in understanding WBS.

I don’t think we’ve even thought about [PLE perspectives] when we were having these discussions, so this is eye opening …

— Medical Personnel

“I was surprised to hear that [PLE] wanted to know the wastewater testing, what the results showed, because – and I’m thinking about it, what was that information going to do for them, right?”

— Carceral Personnel

Others stated that they’ve heard similar reflections (Brown, 2024) on COVID protocols from residents informally.

I think it’s quite in line with a lot of what we’ve heard on the compound. And when you do speak to patients… they do tell you [their experience with mitigation protocols.]

— Carceral Personnel

CP voiced appreciation in receiving feedback from PLE and said they understood some of the frustrations expressed. CP emphasized their commitment to the health and safety of residents above all else. When they were asked about what they wanted to tell PLE, they offered various messages reinforcing commitment to resident safety and health.

“We understand that this has been difficult for you but we’re doing the best we can, and ultimately we’re just trying to keep everyone here safe.”

—Medical Stakeholder

“We don’t want them to look at it [the mitigation protocols] as a punitive effect. It is simply something to make sure that you leave as healthy as you came or better than you came if possible.”

— Medical Stakeholder

Some CP volunteered that although not all concerns of PLE could be addressed, there was some merit in hearing them, and integrating them where possible could be beneficial. Others insisted that PLE’s desire to know WBS results and processes should not preclude decision-makers from withholding WBS information. The greater interest of the jail community was prioritized across all CP input.

“We can’t always accommodate every request, but it’s useful to hear what [PLE are] experiencing, what they have to say, and then we can incorporate it, you know, in the best interest of everybody.”

— Medical Stakeholder

“We certainly appreciate the detainees’ perspectives on what we’re doing, but we wouldn’t let that dictate our decisions, you know, what we think would be in the best interest of the – you know, the health of the entire jail compound.”

— Medical Stakeholder

### b. Mechanisms of Collaboration between Persons with Lived Experience of Incarceration and Carceral Personnel

When asked about collaboration, PLE found it difficult that they could not directly communicate with CP within the scope of this project. According to some PLE, never communicating directly with CP hindered true partnership. This communication structure, along with the sentiments that CP were not genuine in their messaging to PLE, reinforced the impression that PLE felt of being left out of decision-making by CP while incarcerated.

“It wasn’t like we were talking to them [leadership]. It was like you guys [researchers] were the go between, you know? That’s not really a direct partnership, though.”

—PLE

“[CP] would talk to you guys differently than they would talk to us if we were talking to them about it.”

—PLE

“Most of the staff don’t have nothing to do with us. The only time they have something to do with us is when it’s a problem…They’ll talk to you. They’ll help you. They’ll give you stuff, but other than that, they don’t associate with us. It’s we govern our self in there.”

—PLE

In discussions among CP, the notion of incorporating PLE feedback generated further exchange about existing avenues of communication with incarcerated persons. No carceral leadership or staff from any site reported mechanisms in place in which resident feedback specific to mitigation measures, such as quarantine and isolation, could be collected. Feedback at some jails was traditionally communicated informally through medical patient-facing staff.

“I’m the arms and legs in the jail, so typically what happens when I go to a unit and such to talk to the patients… They raise some of the concerns, I’m the one who brings …this to the management…there is not any set up system for that kind of comment to deliver [messages from residents] to the management.

—Medical Stakeholder

In FGDs, CP brainstormed ways current systems could be adapted to fill this gap. One medical stakeholder proposed expanding the scope of internal advisory councils to include mitigation and community health-related feedback.

“We don’t really have a formal thing in place right now [to incorporate feedback from residents when crafting infection control strategies] … but we’ve been trying to build out these internal advisory councils at [jail], and we’ve been able to launch them in one area. And the whole point is to really bring staff and people incarcerated together to discuss any issues. So, I would think as we continue to formulate those that would be an opportunity for where we could incorporate ways to get this type of feedback.”

—Medical Stakeholder

Additionally, the grievance system, which is utilized in some form at each jail, was also identified as a possible resource that could be adapted to include infection control feedback. Currently, residents use the grievance process to submit generalized complaints rather than concerns specific to IC.

“We continue to have a grievance process whereby those in our custody could voice concerns about any issue.”

—Medical Stakeholder

Some medical stakeholders appreciative of the feedback felt the setting of a focused meeting or an independent system, rather than an adapted one, would be preferred. Citing that dedicated time to hearing resident feedback could provide more relatable messages in a different setting.

“From the day-to-day provider’s perspective, it’s always nice to have these comments coming outside of the practice day to day things, because even though I fully agree what I’ve heard of the comments, sometimes when you are under the stress of the work these are kind of filtered through, when you hear in different settings like this, like I can relate more. So again, like incorporation is probably important but at the same time having this separate timing to share this type of information and comments is really helpful for the providers.”

— Medical Stakeholder

PLE offered a variety of solutions for communications with CP. Overall, the theme emerged of a desire to have an entity representative of jail residents to facilitate information sharing and feedback. Not everyone agreed on what this should look like; some were in support of an incarcerated representative, while others believed that a neutral third party would be more effective. Some believed an incarcerated representative would have the direct experience to give useful representation. Others believed that due to the diversity of residents, a third party would be more effective in representing the needs of the many types of residents.

“It might be kind of hard because you got, you know, a variety of inmates… So not everybody going to see eye to eye, but the person, whoever is voted in should be, you know, impartial to all of that, you know what I mean? Non-judgmental… And just like the overall, you know, humanization of people in general.”

—PLE

“I would think it would have to be like a third party… like a human rights group”

— PLE

“I was thinking … if they had one voice for the whole body of inmates, you know what I mean?… that person is voted in to speak on behalf of everybody.”

— PLE

Other PLE touched on the creation of a new grievance system. PLE agreed that the current grievance systems were difficult to access. A suggestion was offered around a grievance kiosk specifically for health concerns.

“To implement this system the jails could create a feedback form that the residents can fill out anonymously. The form should include questions about cleanliness of the facility, availability of hand sanitizer and soap and access to masks and other protective equipment.”

— PLE

Our findings suggest the collaboration between CP and PLE as stakeholders in jointly undertaking infection control was fruitful, though a new experience for both parties. Although some CP questioned the utility of including team members who were not decision-makers in implementation activities, the process of including diverse stakeholders helped identify barriers to collaboration within staffing structures and residents. Additionally, perspectives of PLE were recognized as useful to infection control to such a degree that CP voiced support of a sustainable method of receiving mitigation feedback.

### Quantitative Results: Research Engagement Survey Tool

To further explore the dynamics of collaboration found in our qualitative analysis, we used data from the REST to quantify the differing level engagement. There were 19 total respondents of the REST at the conclusion of the project, 12 of whom were CP and 7 of which were PLE. We report a summary of EP-specific scores and overall scores in Table 3 and Appendix Table 4.

EP-specific scores for quality and quantity show that CP reported higher scores for each measured EP, resulting in an overall higher score compared to PLE on the quality scale (CP _Quality Avg(SD)_= 3.9 (0.7) vs PLE _Quality Avg(SD)_=3.2 (1.0) and quantity scale (CP _Quantity Avg(SD)_= 4.1(0.5) vs PLE _Quantity Avg(SD)_=3.3 (1.0) (Table 3).

**Figure 2.**
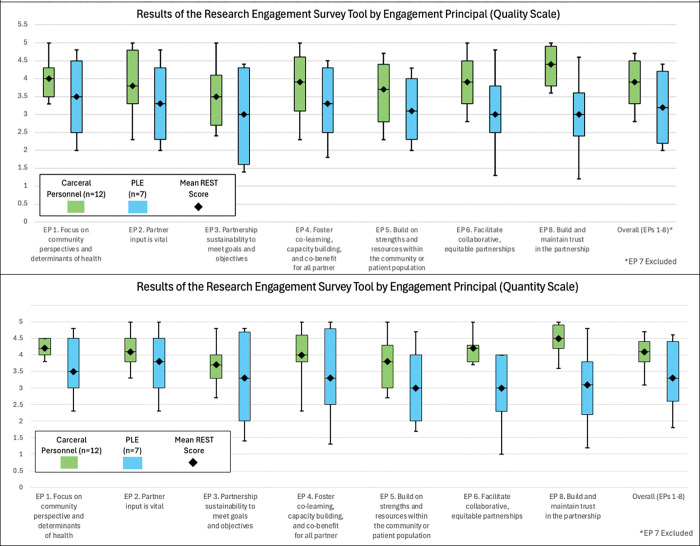
Graphical Results of the Research Engagement Survey Tool for Carceral Personnel vs Persons with Lived Experience of Incarceration Stakeholder Team Members n=19

The greatest differences in engagement between CP and PLE are presented in EPs 6 and 8, which address equitable partnership and trust between partners respectively (Figure 2).

## Discussion

The CRAINES jail-based WBS implementation project provided an opportunity to involve PLE in a multi-stakeholder, mixed-methods study which illustrated the many challenges associated with stakeholder involvement in carceral health. Few other sectors in public health have had less participatory involvement and greater need for PLE engagement in research.

Collaboration between diverse carceral stakeholders including PLE provided lessons on the intricacies of carceral policymaking. Initially, many CP were unclear on the utility of including PLE on WBS implementation teams. However, as the partnership with PLE continued to develop, CP not only empathized with the experience of living through enacted infection control policies but also recognized the perspectives of PLE as useful to improving infection control systems. Furthermore, in response, CP brainstormed ways in which such feedback from incarcerated persons could be delivered more routinely and systematically by adapting processes specific to their jail.

Reflection on the collaborative processes between decision-makers, leadership, and staff in the jail were highlighted throughout FGDs. The barriers in collaboration included staffing structures that emphasize a top-down decision-making approach. Medical stakeholders recognized the benefit of engaging staff, such as facilities management who would be responsible for the routine collection of wastewater as part of WBS. There was also recognition of leveraging the relationship between residents and the medical staff who interface with them commonly. Findings show this as an informal method of relaying mitigation control feedback to medical leadership.

According to PLE, one of the things that hindered collaboration was the indirect communication between carceral stakeholders. This challenge was due, in part, to the study design. Good research practice for FGDs is to avoid power imbalances—participants are meant to have similar backgrounds (Patton, 2015), justifying our communication structure in this project of separating PLE and CP in project activity. Illustrating this point, 5 of 8 PLE participants returned to jail after their index incarceration. Returning to the jail where the decision-makers worked introduced an immense power imbalance. Future work may need to utilize different strategies in communication between stakeholders.

The selection of diverse stakeholders also presented an opportunity to test how well the REST instrument could measure differing degrees of engagement. The mean response for PLE in every domain tested consistently fell below that of CP on both the quality and quantity scales. This finding was consistent with the qualitative results, in which PLE expressed dissatisfaction with the elements of the partnership. The greatest difference in engagement between PLE and CP were illustrated in EPs 6 and 8 which address equitable partnership and trust between partners, respectively. This finding aligns with key qualitative findings in this manuscript and in previous work where PLE emphasize trustworthiness in health messaging (Brown, 2024).

Such differences in REST scores found in this project reinforce the importance of examining REST scores between stakeholder roles, which can highlight important differences in engagement. Currently, there is only one other publication that utilizes REST in a similar stratified fashion(Hoke A.M., 2023).

There were limitations in the qualitative assessment of stakeholder engagement. Per the project design, the study team took on the role of dissemination. Such role excluded the role of any implementation team members for EP 7, resulting in overall REST scores not factoring in EP 7. The exclusion of EP 7 thus yields unvalidated results per the validation process of the REST. This study was also limited by small sample size, which did not allow for sufficient power to detect statistical differences between carceral personnel and PLE REST scores.

Moreover, this project involved only 4 out of over 2800 jails across the country and all were medium to mega-sized jails; none were rural. The PLE who participated were a convenience sample chosen by site leads. Because of competing demands and rapid turnover of staff, the number of FGD and interviews was determined according to feasibility, rather than a formal discussion of whether saturation had been reached.

Going forward, recognizing both the disparity PLE reported in both the quality and quantity of engagement and the inherent power imbalance in carceral setting, studies may need to thoughtfully plan collaboration efforts in which PLE have greater agency in project activity.

We encourage the development of a safe channel where the carceral residents can freely communicate their perspective on infection control mitigation policies. Carceral personnel found such feedback useful as part of this project and hoped to establish a routinely employed feedback system for continuous quality improvement of infection control protocols, when allowable. Facilitating communication through feedback systems may help dispel false or negative notions and increase support for meaningful collaborations across carceral systems.

## Supplementary Material

Supplementary Files

This is a list of supplementary files associated with this preprint. Click to download.

• Appendix.docx

## Figures and Tables

**Table 1.** Demographics of PLE Who Were Jailed at Enrolled Facilities During the Pandemic n=8

		Count (%)
Race		
Black		7 (87.5%)
White		1 (12.5%)
Gender		
Male		7 (87.5%)
Education Level		
< High School completion or GED Equivalent		0 (0%)
High School or GED completion		3 (37.5%)
Some College		3 (37.5%)
College Completion		1 (12.5%)
Advanced Degree		0 (0%)
Missing		1 (12.5%)
Length of Stay in Jail(s) During COVID-19 Pandemic		
1–6 months		2 (25.0%)
7–12 months		3 (37.5.0%)
>12 months		2 (25.0%)
Missing		1 (12.5%)
Reincarcerated During Duration of Project		
Yes		5 (62.5%)
No	2 (25.0%)	
Missing	1 (12.5%)	
Avg Age at time of enrollment, years		38.4 (24.0–52.0)
Missing n=1		

**Table 2.** Domains of Research Engagement Survey Tool

EP1.	Focus on community perspectives and determinants of health
EP2.	Partner input is vital
EP3.	Partnership sustainability to meet goals and objectives
EP4.	Foster co-learning, capacity building, and co-benefit for all partners
EP5.	Build on strengths and resources within the community or patient population
EP6.	Facilitate collaborative, equitable partnerships
EP7.	Involve all partners in the dissemination process
EP8.	Build and maintain trust in the partnership

**Table 3.** Results of the Research Engagement Survey Tool for All Implementation Team Members, n=19

		*All (n=19)*				*PLE (n=7)*				*Carceral Personnel (n=12)*			
*Quality:* *How Well EPs were Accomplished*		Mean (SD)	Range	Q1	Q3	Mean (SD)	Range	Q1	Q3	Mean (SD)	Range	Q1	Q3
	Engagement Principle (EP)										
	1. Focus on community perspectives and determinants of health	3.8 (0.8)	2.0–5.0	3.3	4.3	3.5 (1.1)	2.0–4.8	2.5	4.5	4.0 (0.5)	3.3–5.0	3.5	4.3
	2. Partner input is vital	3.6 (0.9)	2.0–5.0	3.0	4.3	3.3 (1.0)	2.0–4.8	2.3	4.3	3.8 (0.8)	2.3–5.0	3.3	4.8
	3. Partnership sustainability to meet goals and objectives	3.4 (1.0)	1.4–5.0	2.7	4.2	3.0 (1.2)	1.4–4.4	1.6	4.3	3.5 (0.8)	2.4–5.0	2.7	4.1
	4. Foster co-learning, capacity building, and co-benefit for all partners	3.7 (1.0)	1.8–5.0	2.8	4.5	3.3 (1.0)	1.8–4.5	2.5	4.3	3.9 (0.9)	2.3–5.0	3.1	4.6
	5. Build on strengths and resources within the community or patient population	3.4 (0.9)	2.0–4.7	2.7	4.3	3.1 (0.9)	2.0–4.3	2.3	4.0	3.7 (0.8)	2.3–4.7	2.8	4.4
	6. Facilitate collaborative, equitable partnerships	3.5 (1.0)	1.3–5.0	2.8	4.3	3.0 (1.1)	1.3–4.8	2.5	3.8	3.9 (0.8)	2.8–5.0	3.3	4.5
	8. Build and maintain trust in the partnership	3.9 (1.0)	1.2–5.0	3.6	4.6	3.0 (1.1)	1.2–4.6	2.4	3.6	4.4 (0.6)	3.6–5.0	3.8	4.9
												
	Overall (Eps 1–6, 8)	3.6 (0.8)	2.0–4.7	2.8	4.4	3.2 (1.0)	2.0–4.4	2.2	4.2	3.9 (0.7)	2.8–4.7	3.3	4.5

		*All (n=19)*				*PLE (n=7)*				*Carceral Personnel (n=12)*			
Quantity: How Often Eps were Accomplished		Mean	Range (SD)	Q1	Q3	Mean	Range (SD)	Q1	Q3	Mean (SD)	Range	Q1	Q3
	Engagement Principle (EP)												
	1. Focus on community perspectives and determinants of health	3.9 (0.7)	2.3–4.8	3.8	4.5	3.5 (0.9)	2.3–4.8	3.0	4.5	4.2 (0.3)	3.8–4.5	4.0	4.5
	2. Partner input is vital	4.0 (0.7)	2.3–5.0	3.8	4.5	3.8 (1.0)	2.3–5.0	3.0	4.5	4.1 (0.5)	3.3–5.0	3.8	4.5
	3. Partnership sustainability to meet goals and objectives	3.5 (0.9)	1.4–4.8	2.8	4.2	3.3 (1.3)	1.4–4.8	2.0	4.7	3.7 (0.7)	2.7–4.8	3.3	4.0
	4. Foster co-learning, capacity building, and co-benefit for all partners	3.8 (1.0)	1.3–5.0	3.3	4.8	3.3 (1.3)	1.3–5.0	2.5	4.8	4.0 (0.8)	2.3–5.0	3.8	4.6
	5. Build on strengths and resources within the community or patient population	3.5 (1.0)	1.7–5.0	2.7	4.0	3.0 (1.1)	1.7–4.7	2.0	4.0	3.8 (0.8)	2.7–5.0	3.0	4.3
	6. Facilitate collaborative, equitable partnerships	3.6 (1.0)	1.0–5.0	3.3	4.1	3.0 (1.1)	1.0–4.0	2.3	4.0	4.2 (0.5)	3.7–5.0	3.8	4.3
	8. Build and maintain trust in the partnership	4.0 (1.0)	1.2–5.0	3.6	4.8	3.1 (1.2)	1.2–4.8	2.2	3.8	4.5 (0.4)	3.6–5.0	4.2	4.9
	Overall (Eps 1–6, 8)	3.8 (0.8)	1.8–4.7	3.1	4.4	3.3 (1.0)	1.8–4.6	2.6	4.4	4.1(0.5)	3.1–4.7	3.8	4.4

## Data Availability

The data analyzed in this study are included in this [published] article. As part of the consent process, to maintain confidentiality, the participants were promised that identifying information would not be released. For this reason, we are unable to release raw qualitative data without redaction such as transcripts. Researchers may request elements of the collected data from the corresponding author upon reasonable request
